# New Invertebrate Vectors of Okadaic Acid from the North Atlantic Waters—Portugal (Azores and Madeira) and Morocco

**DOI:** 10.3390/toxins7124885

**Published:** 2015-12-08

**Authors:** Marisa Silva, Inés Rodriguez, Aldo Barreiro, Manfred Kaufmann, Ana Isabel Neto, Meryem Hassouani, Brahim Sabour, Amparo Alfonso, Luis M. Botana, Vitor Vasconcelos

**Affiliations:** 1 Department of Biology, Faculty of Sciences, University of Porto, Rua do Campo Alegre, 4619-007 Porto, Portugal; marisasilva17@gmail.com (M.S.); aldo.barreiro@gmail.com (A.B.); 2 Interdisciplinary Center of Marine and Environmental Research–CIMAR/CIIMAR, University of Porto, Rua dos Bragas 289, 4050-123 Porto, Portugal; mkaufmann@ciimar.up.pt (M.K.); aneto@uac.pt (A.I.N.); 3 Department of Pharmacology, Faculty of Veterinary, University of Santiago of Compostela, 27002 Lugo, Spain; ines.rodriguez.filgueiras@usc.es (I.R.); amparo.alfonso@usc.es (A.A.); Luis.Botana@usc.es (L.M.B.); 4 University of Madeira, Marine Biology Station of Funchal, 9000-107 Funchal, Madeira Island, Portugal; 5 Center of Interdisciplinary Marine and Environmental Research of Madeira—CIIMAR-Madeira, Edifício Madeira Tecnopolo, Caminho da Penteada, 9020-105 Funchal, Madeira, Portugal; 6 Department of Marine Biology, University of Azores, 9501-801 Ponta Delgada, Azores, Portugal; 7 Phycology Research Unit—Biotechnology, Ecosystems Ecology and Valorization Laboratory, Faculty of Sciences El Jadida, University Chouaib Doukkali, BP20 El Jadida, Morocco; hassouani@hotmail.com (M.H.); sabour.b@ucd.ac.ma (B.S.)

**Keywords:** okadaic acid, new vectors, Madeira Island, São Miguel Island, Morocco

## Abstract

Okadaic acid and its analogues are potent phosphatase inhibitors that cause Diarrheic Shellfish Poisoning (DSP) through the ingestion of contaminated shellfish by humans. This group of toxins is transmitted worldwide but the number of poisoning incidents has declined over the last 20 years due to legislation and monitoring programs that were implemented for bivalves. In the summer of 2012 and 2013, we collected a total of 101 samples of 22 different species that were made up of benthic and subtidal organisms such echinoderms, crustaceans, bivalves and gastropods from Madeira, São Miguel Island (Azores archipelago) and the northwestern coast of Morocco. The samples were analyzed by UPLC-MS/MS. Our main objective was to detect new vectors for these biotoxins. We can report nine new vectors for these toxins in the North Atlantic: *Astropecten aranciacus*, *Arbacia lixula*, *Echinaster sepositus*, *Holothuria sanctori*, *Ophidiaster ophidianus*, *Onchidella celtica*, *Aplysia depilans*, *Patella* spp., and *Stramonita haemostoma*. Differences in toxin contents among the species were found. Even though low concentrations were detected, the levels of toxins that were present, especially in edible species, indicate the importance of these types of studies. Routine monitoring should be extended to comprise a wider number of vectors other than for bivalves of okadaic acid and its analogues.

## 1. Introduction

**Figure 1:**
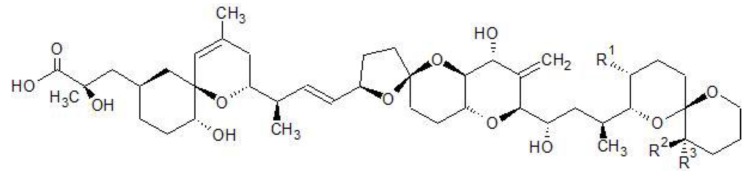
Chemical structures of OA, DTX1&2.

Diarrheic Shellfish Poisoning (DSP) is a syndrome caused by the ingestion of organisms contaminated with the phosphatase inhibitors group of okadaic acid (OA) and its analogs; dynophysistoxin 1 and 2 (DTX1, DTX2) (Figure 1). These diarrheic shellfish toxins (DST) were first isolated from two sponge species: *Halichondria okadai* and *H. melanodocia* [1,2], and are mainly produced by dynoflagellates of the genera *Dynophysis*, *Phalacroma*, and *Prorocentrum* [3,4,5]. Regarding the mechanism of action, these toxins are strong inhibitors of serine/threonine phosphatases, especially types 1 and 2A with a particularly high affinity to 2A [6,7]. This inhibition results in the increase of the phosphorylation of a number of proteins leading to significant cell alterations, being OA and DTX1,which were also reported as tumor promotors and inducers of genotoxicity and cytotoxicity at low concentrations in marine invertebrates [8,9].

**Table:** 

Toxin	R^1^	R^2^	R^3^
OA	CH_3_	H	H
DTX1	CH_3_	CH_3_	H
DTX2	H	H	CH_3_

Although first reported in Japan, poisoning incidents occur all over the world and the most common route of intoxication is via ingestion of contaminated fish and shellfish [10,11,12,13]. These phycotoxins are heat and frost resistant and the taste and odor of the contaminated organisms remain unchanged [14,15,16]. DSP is known for its serious gastrointestinal symptoms, from chills to diarrhea, and the severity of the intoxication depends on the amount of toxin that the patient was exposed to [17].

Given their global incidence, DSTs are regulated worldwide. Studies on mice, together with epidemiologic studies, led to the establishment of the Lowest Observed Effect Level (LOEL) of 50 μg OA equivalents/person [18]. Currently, the toxic equivalent factors (TEF) have been established, (Table 1), and the limit value for the European Union is 160 µg OA equivalents/ kg shellfish meat (SM) [18,19]. Regarding detection methods, the European Food Safety Agency (EFSA) recommends the use of analytical techniques such as LC-MS/MS [18]. This recommendation was later reinforced in 2011 by the European Commission (EC) suggesting that this technique should be established as a reference method for DST detection by 31 December 2014 [20]. Analytical procedures are more reliable, do not have the ethical issues that come with the use of mice bioassay (MBA) technique, are able to identify several toxins in a mixture with high degree of sensitivity.

**Table 1:** Toxic equivalent factors for OA and its analogs.

Toxin	TEF	Reference
OA	1	[18]
DTX1	1	[18]
DTX2	0.6	[18]

Prior research demonstrated that DSTs can be found in unusual vectors along the food chain, although monitoring for this group of toxins, exclusively in bivalves, is simplistic and underestimates the risk to public health [21]. This argument is reinforced by the evidence that OA is bioaccumulated through the food web [21]. In this study, we surveyed the Portuguese islands of Madeira (Madeira archipelago) and São Miguel (Azores archipelago), and the northwestern coast of Morocco for new vectors through intertidal and SCUBA diving harvesting. We collected 22 species of benthic organisms including gastropods (sea-snails, sea-slugs, and limpets), bivalves (mussels), crustaceans (barnacles), and echinoderms (starfishes, sea-urchins, and sea-cucumbers). The fact that most of the above-mentioned species are edible and commercially important species was a determining factor in the selection of these species (Table 2). Inedible species were also sampled for their importance in the food chain. We believe that our data contribute to the development and updating of legislation regarding the monitoring procedures of these toxins in order to better protect public health.

**Table 2:** Species sampled and their trophic level, average number of specimens comprising a pooled sample (AvNr), and number of samples collected (NrP Samples)—from Madeira in September 2012, São Miguel Island, Azores, in June 2013, and Morocco in July 2013— edibility and monitoring status (M. status). Availability of animals is dependent on their geographical distribution and ecology.

Species	Trophic Level	Sampling Site(s)	Nr P Samples	AvNr	Edible	M. Status	Ref.
*Astropecten aranciacus*	2nd level predator	Madeira	1	2	No	No	[22]
*Echinaster sepositus*	2nd level predator	Madeira	1	3	No	No	[23]
*Marthasterias glacialis*	2nd level predator	Madeira/Azores/Morocco	8	1	No	No	[24]
*Ophidiaster ophidianus*	Detritivorous	Madeira/Azores	5	1	No	No	[23]
*Paracentrotus lividus*	Grazer	Madeira/Azores/Morocco	7	1	Yes	No	[25]
*Diadema africanum*	Grazer	Madeira	2	1	No	No	[26]
*Sphaerechinus granularis*	Grazer	Azores	4	1	Yes	No	[27]
*Arbacia lixula*	Grazer	Madeira/Azores/Morocco	9	4	No	No	[28]
*Holothuria(Platyperona)sanctori*	Deposit feeder	Morocco	4	1	Yes	No	[29,30]
*Pollicipes pollicipes*	Filter feeder	Morocco	3	35	Yes	No	[24]
*Monodonta lineata*	Grazer	Morocco	5	86	Yes	No	[31]
*Onchidella celtica*	Grazer	Morocco	1	50	No	No	[32]
*Pattela aspera*	Grazer	Madeira	2	15	Yes	No	[24]
*Patella* spp*.*	Grazer	Morocco	4	12	Yes	No	[24]
*Pattela candei*	Grazer	Azores	3	10	Yes	No	[24]
*Umbraculum umbraculum*	Grazer	Madeira	1	1	No	No	[33]
*Stramonita haemostoma*	2nd level predator	Madeira/Azores/Morocco	5	15	No	No	[34]
*Charonia lampas*	3rd level predator	Madeira/Morocco	3	1	Yes	No	[35]
*Cerithium vulgatum*	Grazer	Morocco	1	40	Yes	No	[36]
*Gibbula umbilicalis*	Grazer	Morocco	3	100	Yes	No	[31]
*Mytilus* spp*.*	Filter feeder	Morocco	4	30	Yes	Yes	[37]

## 2. Results and Discussion

**Figure 2:**
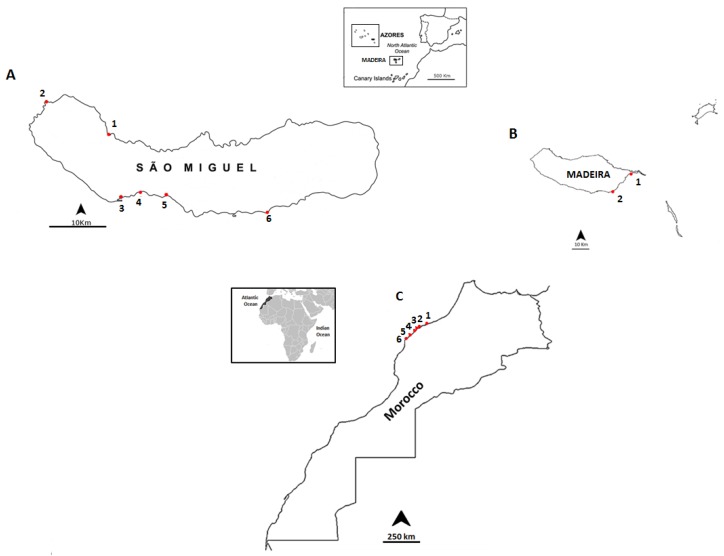
Location of the sampling points: (**A**) the coast of São Miguel Island, Azores archipelago: 1-Cruzeiro; 2-Mosteiros; 3-Étar; 4-São Roque; 5-Lagoa; and 6-Caloura; (**B**) the coast of Madeira: 1-Reis Magos; 2-Caniçal; and (**C**) the northwestern coast of Morocco: 1-Casablanca Corniche; 2-El Jadida Haras; 3-El Jadida Sâada; 4-Sidi Bouzid; 5-Mrizika; and 6-Oualidia.

In this study, a total of 101 samples were collected from three different sampling sites:

Madeira (25 samples) in September 2012, São Miguel Island, Azores (37 samples), in June 2013 and the northwestern coast of Morocco (39 samples) in July 2013 (Figure 2).

Several species belonging to distinct taxa were collected, comprising starfish (*A. aranciacus*, *E. sepositus*, *M. glacialis*, *O. ophidianus*), sea-urchins (*A. lixula*, *D. africanum*, *P. lividus*, *S. granularis*), sea-cucumber (*H. sanctori*), crustaceans (*P. pollicipes*), bivalves (*Mytillus* spp.) and gastropods (*A. depilans*, *C. vulgatum*, *C. lampas*, *G. umbilicalis*, *M. lineata*, *O. celtica*, *Patella* spp., *P. candei*, *P. tenuis tenuis*, *P.aspera*, *S. haemostoma*, and *U. umbraculum*).

**Figure 3:**
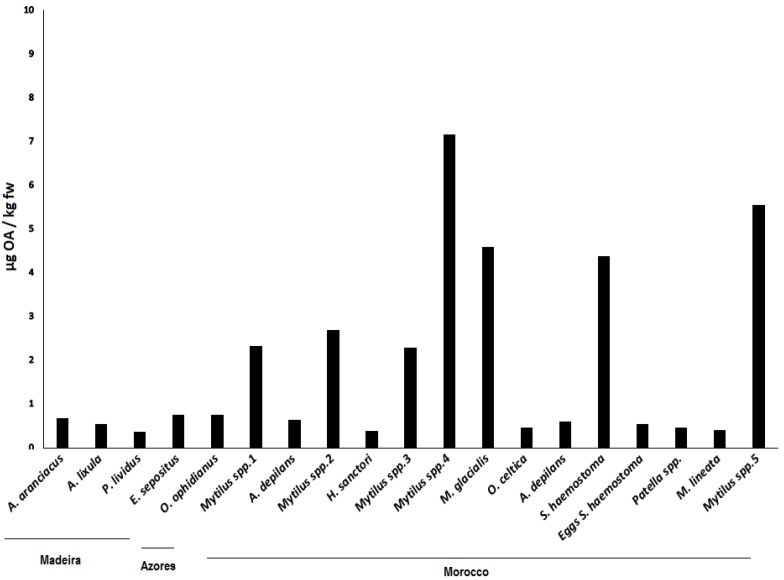
OA (µg/Kg fresh weight (fw)) for all sampled groups of organisms in Madeira (Starfish—*A. aranciacus*, *E. sepositus*; Sea-urchins—*A. lixula*, *P. lividus*) Azores (Starfish—*O. ophidianus*) and Morocco (Starfish—*M. glacialis*; Sea-cucumbers—*H. sanctori*; Gastropods—*A. depilans*, *O. celtica*, *S. haemostoma*, *Patella* spp., *M. lineata*; Bivalves- *Mytilus* spp.).Numbers correspond to different sampling locations in Morocco: 1-Casablanca Corniche; 2-Sidi Bouzid; 3-El Jadida Saâda; 4-Mrizika; and 5-Oualidia.

OA and its analogues were screened in the three sampling sites. A total of 19% of the samples contained quantifiable OA contents for OA (above the limit of quantification (LOQ)), but neither DTX1 nor DTX2 were detected (Figure 3). DTX3 was also screened in all of the samples but was below the limit of detection (LOD) of the equipment. The LOD and LOQ for AO/DTX1/DTX2 in our equipment were 0.468 ng/mL and 1.56 ng/mL, respectively. In order to analyze and quantify OA/DTX1/DTX2 in the samples, a 50× step was added whereby the amounts of 1.85–35 ng/mL (0.37–7 µg/Kg) were detected. Following the official control method of the European Union, alkaline hydrolysis was performed in order to detect the esoteric forms of OA and DTX toxins. Within this method, a ratio of NaOH/HCl and a sample extract is required to maintain pH conditions and cannot be modified. For this reason, the samples cannot be concentrated. In order to maintain this ratio, the samples were diluted (50× dilution) and hydrolysis was done following the procedure. While the hydrolyzed samples were being analyzed, the diluted control without hydrolysis was also checked. In both samples, hydrolyzed and unhydrolyzed, OA could be detected in the samples with a higher concentration, but could not be quantified. This small amount was above the LOD but below the LOQ. No changes were observed in these peaks before and after hydrolysis. If the samples had been concentrated, the pH of the solution would have changed and the toxins could have degraded.

The majority of the samples with OA (73.7%), as well as the samples with the highest concentrations, were detected on the coast of Morocco, followed by Madeira (21.1%). This might be due to the fact that both sampling sites are at the same latitude. For São Miguel Island (Azores), only one measurable sample was detected in the starfish *O. ophidianus*.

Average concentrations detected were all below the current limit implemented in the European legislation—160 µg OA equivalents/ kg shellfish meat [18]—and ranged from 0.368 µg/kg fresh weigh (fw), in *P. lividus*, to 7.157 µg/kg fw in *Mytilus* spp.

Regarding statistics, the first step of the gamma hurdle model was a Generalized Linear Model (GLZ) performed with the data of presence/absence of OA, using binomial distribution error.

This model was applied separately to each geographical location. The results of the model’s analysis of deviance as well as the coefficients rescaled to a logistic probability [0,1] are shown in Table 3. The “organism” did not turn out to be a significant factor, most likely due to the low number of samples with quantifiable OA contents, except in Morocco. In Morocco, the highest probability corresponded to the bivalve, whereas all other organisms had very low probabilities of containing OA. The second part of the model with the gamma error distribution analyzes the variation in OA concentration, showing quantifiable results among those samples. It was not possible to perform this analysis in Azores because there was only one single sample containing OA. The factor “organism” was significant both in Madeira and Morocco (Table 4).In Madeira, OA only appeared in sea urchin and star fish, with star fish containing three times more OA, on average (1.58 *versus* 0.45, Table 4). In Morocco, the bivalves contained much more OA than all the other organisms, the closest one being the sea star, with an average of approximately ¼ of bivalve OA content (4 *versus* 1.15, Table 4).

**Table 3:** Results of the binomial regression model for OA occurrence with “organism” as a factor.

Analysis of Deviance				
Location	Factor	χ*^2^*	*df*	*p*
Madeira	Organism	3.9	3	0.28
	Rescaled model coefficients: sea urchin = 0.16; star fish = 0.71; gastropod = 1.6 × 10^−8^; limpet = 1.6 × 10^−8^			
Azores	Organism	2.1	3	0.56
	Rescaled model coefficients: sea urchin = 4.3 × 10^−10^; star fish = 1; gastropod = 0.5; limpet = 0.5			
Morocco	Organism	19.8	7	<0.01
	Rescaled model coefficients: bivalve = 1; crustacean = 1 × 10^−17^; sea urchin = 1 × 10^−17^; star fish = 3.2 × 10^−9^; gastropod = 1.1 × 10^−9^; limpet = 1.1 × 10^−9^; sea snail = 6.4 × 10^−9^; sea cucumber = 1.1 × 10^−9^			

**Table 4:** Results of the gamma regression model for OA occurrence with organism as factor.

Analysis of Deviance				
Location	Factor	χ*^2^*	*df*	*p*
Madeira	Organism	5.4	1	<0.05
	Rescaled model coefficients: sea urchin = 0.45; star fish = 1.58			
Morocco	Organism	12.8	5	<0.05
	Rescaled model coefficients: bivalve = 4; star fish = 1.15; gastropod = 0.44; limpet = 0.12; sea snail = 0.15; sea cucumber = 0.1			

The high number of positive detections in Morocco could be explained by an increased eutrophication effect, due to larger population density, continental runoff, and industrial pollution. In comparison to the previous study, the amounts of OA detected are quite different, ranging from 0.58 µg/Kg fw to 429.41 µg/Kg fw [21], even though the number of screened species is higher in the present study. Here, the higher concentrations that were detected could equally be due to higher anthropogenic inputs, although they were detected in different taxa, namely gastropods [21]. From a statistical point of view, it is not possible to compare the bioaccumulation results between the studies due to the lower positive hits obtained in the present study.

Owing to the oligotrophic waters of Madeira and Azores archipelagos, bivalves are not common, which makes gastropods and echinoderms a good alternative for the monitoring of OA and its derivatives in both archipelagos.

We report nine new vectors for OA in the species *A. aranciacus*, *A. lixula*, *A. depilans*, *E. sepositus*, *H. sanctori*, *O. celtica*, *O. ophidianus*, *Patella* spp., and *S. haemostoma*. We also detected OA in the eggs of *S. haemostoma* indicating a potential parental transfer of the toxin to the offspring. These kind of cases have already been reported, as an example in *Takifugo rubripes* larvae that are protected by maternal tetrodotoxin [38].

## 3. Experimental Section

### 3.1. Selected Species and Sampling Sites

The coasts of the Portuguese islands of Madeira (Madeira archipelago), São Miguel (Azores archipelago), and the northwestern coast of Morocco, were surveyed for non-traditional vector species for Okadaic Acid and its analogs. These locations were chosen as a result of collaborations and projects with the local entities, who also allowed us to survey these areas.

Several edible and non-edible species were selected (*n* = 22) to search for potential new vectors and the prevalence of the screened biotoxins in the food web: gastropods (*Patella tenuis tenuis*, *Patella aspera*, *Stramonita haemostoma*, *Umbraculum umbraculum*, *Charonia lampas*, *Patella candei*, *Patella* spp., *Aplysia depilans*, *Monodonta lineata*, *Cerithium vulgatum*, *Gibbula umbilicalis*,*Onchidellaceltica*), crustaceans (*Pollicipes pollicipes*), bivalves (*Mytillus* spp.), starfish (*Astropecten aranciacus*, *Ophidiaster ophidianus*, *Marthasterias glacialis*, *Echinaster sepositus*), sea-cucumber (*Holothuria (Platyperona) sanctori*), and sea-urchins (*Paracentrotus lividus*, *Arbacia lixula*, *Sphaerechinus granularis*, *Diadema africanum*). Benthic organisms were harvested from the intertidal areas during low tide and by scuba diving expeditions: the island of Madeira was surveyed in September 2012, São Miguel Island, Azores, and the Moroccan coast were sampled in June and July 2013, respectively (sampling sites are displayed in Table 5). Two samples of *Patella tenuis tenuis* and *P. aspera* were purchased at local markets in Madeira, which were caught off the northern coast of the island (32°51ʹ17.02ʹʹ N; 17°01ʹ54.02ʹʹ W). Sample identification was aided by the use of field guides. Organisms were transported to the laboratory in refrigerated containers. Samples were frozen at −20 °C, if they were not processed immediately.

**Table 5:** Sampling sites and respective geographical coordinates, surveyed during September of 2012 and June and July of 2013.

Date	Location	Sampling Site	Geographic Coordinates
September 2012	Madeira Island	Reis Magos	32°39ʹ16.21ʹʹ N; 16°49ʹ05.29ʹʹ W
		Caniçal	32°44ʹ20.08ʹʹ N; 16°44ʹ17.55ʹʹ W
June 2013	São Miguel Island	Cruzeiro	37° 50ʹ31.19ʹʹ N; 25° 41ʹ33.61ʹʹ W
		Étar	37°44ʹ19.31ʹʹ N; 25°39ʹ38.84ʹʹ W
		São Roque	37°45ʹ15.35ʹʹ N; 25°38ʹ31.60ʹʹ W
		Mosteiros	37°53ʹ25.57ʹʹ N; 25°49ʹ14.72ʹʹ W
		Lagoa	37°44ʹ42.38ʹʹ N; 25°19ʹ.47ʹʹ W
		Caloura	37°42ʹ49.34ʹʹ N; 25°29ʹ54.54ʹʹ W
July 2013	Morocco Coast	Casablanca corniche	33°36ʹ01.2ʹʹ N; 7°39ʹ57.5ʹʹ W
		El Jadida Haras	33°14ʹ42.0ʹʹ N; 8°28ʹ37.5ʹʹ W
		El Jadida Sâada	33°14ʹ42.4ʹʹ N; 8°32ʹ26.9ʹʹ W
		Sidi Bouzid	33°13ʹ57.1ʹʹ N; 8°33ʹ20.9ʹʹ W
		Mrizika	32°57ʹ21.8ʹʹ N; 8°46ʹ53.2ʹʹ W
		Oualidia	32°43ʹ55.8ʹʹ N; 9°02ʹ57.6ʹʹ W

### 3.2. Sample Extraction and Hydrolysis Procedure

The Otero *et al.* (2010) extraction protocol was followed [39]. The recuperation rate of the method was calculated with a 95% recovery of OA in mussels. In short, shells were removed when necessary and then animals were homogenized with a blender (A320R1, 700 W, Moulinex, Lisbon, Portugal) in pooled groups in order to obtain 1 g of tissue, with the exception of *Ophidiaster ophidianus*, *Paracentrotus lividus*, *Sphaerechinus granularis*, *Umbraculum umbraculum*, *Diadema africanum*, *Holoturia (Platyperona) sanctori*, *Charonia lampas*, *Marthasterias glacialis*, and *Aplysia depilans*. In these cases, each animal was handled separately since they had enough extractable biomass, so we ended up with a single sample per organism/sampling site. 1 g Homogenized tissue was processed with 3 mL of methanol (Fisher Scientific, Porto Salvo, Portugal), and then centrifuged for 10 min at 2932 g at 4 °C (Centrifugal-Legend RT, Waltham, USA). This procedure was repeated twice and the supernatants were combined and concentrated to dryness (Acid-resistant Centrivap Concentrator, Labconco, Kansas City, USA). Afterwards, residues were re-suspended in 10 mL of water (Milli-Q, Madrid, Spain) and doubly partitioned with dichloromethane (Merck, Darmstadt, Germany). Aqueous layer was discarded and the organic layers (20 mL) were concentrated by drying and re-suspended in 1 mL of methanol. After that, 500 μL was concentrated to dryness, re-suspended in 100 μL of methanol and filtered through a 0.45 μm filter (UltraFree-MC centrifugal devices, Millipore, Bedford, MA, USA). In order to detect and quantify the total content of OA and DTXs, 20 µL of methanolic extract were brought to a final volume of 1 mL. This dilution was hydrolyzed with 125 µL 2.5 M NaOH (Fluka, Sigma-Aldrich, Madrid, Spain), the mixture was homogenized and heated at 76 °C for 40 min. and then cooled to room temperature, neutralized with 125 µL 2.5 M HCl (Panreac, Barcelona, Spain) and homogenized in the vortex [40]. The extract was filtered with 0.45 µm filter and injected 5 µL in the LC column.

### 3.3. Sample Analysis

Analyses were performed using a 1290 Infinity ultra-high-performance liquid chromatography system coupled to a 6460 Triple Quadrupole mass spectrometer (Agilent Technologies, Waldbronn, Germany). Toxin separation was performed with an AQUITY UPLC BEH C18 column (2.1 × 100 mm, 1.7 µm, Waters, Manchester, UK). Column oven was set at 40 °C, samples in the autosampler were cooled to 4 °C and injection volume was 5 µL. Eluent A consisted of 100% water and B acetonitrile (Panreac Quimica, Barcelona, Spain) in water (95:5), both containing 50 mM formic acid (Merck, Madrid, Spain) and 2 mM ammonium formate (Sigma Aldrich, Madrid, Spain). The gradient started with 30%–70% of mobile phase (B) for 3 min, then maintained in 70% B for 4.5 min and decreasing to 30% over 0.1 min and maintained during 1.99 min until the end of the run. Flow rate was 0.4 mL/min.

**Figure 4:**
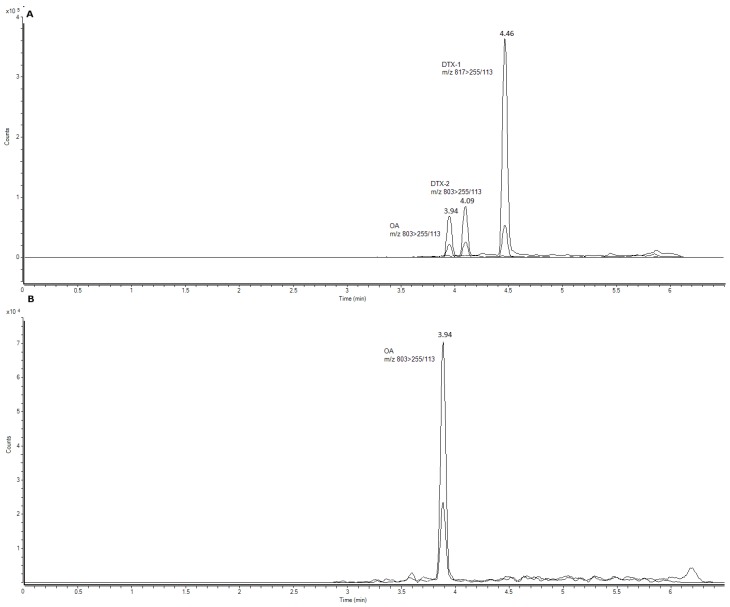
Mass chromatograms of the UPLC-MS/MS obtained under multiple reaction monitoring (MRM) in negative mode. (**A**) Total ion chromatogram (TIC) of OA standard (100 ng/mL), *m*/*z* 803.5 > 255.2/113.2, DTX2 standard (100 ng/mL), *m*/*z* 803.5 > 255.5/113.5 and DTX1 standard (100 ng/mL), *m*/*z* 817.5 > 255.2/113. (B) TIC of a sample with quantifiable OA contents in *Mytillus* spp. (*m*/*z* 803.5 > 255.5/113.5).

MS detection was performed using an Agilent G6460C triple quadrupole mass spectrometer equipped with an Agilent Jet Stream ESI source (Agilent Technologies, Waldbronn, Germany). Source conditions were optimized to achieve the best sensitivity for all compounds. A drying gas temperature of 350 °C and a flow of 8 L/min, a nebulizer gas pressure of 45 psi (Nitrocraft NCLC/MS from Air Liquid, Madrid Spain), a sheath gas temperature of 400 °C and a flow of 11 L/min were used. The capillary voltage was set to 4000 V in negative mode with a nozzle voltage of 0 V and 3500 V. The fragmentor was 260 V and the cell accelerator voltage was 4 V for each toxin in this method. The collision energy, optimized using MassHunter Optimizer software (Agilent Technologies, Waldbronn, Germany), was 52 and 60 eV for OA and DTX2 and 53 and 66 eV for DTX1. The mass spectrometer was operated in multiple reaction monitoring (MRM), detecting in negative mode. Two product ions were analyzed per compound, one for quantification and another for confirmation. The transitions employed were: OA and DTX2 (*m*/*z* 803.5 > 255.2/113.2), DTX1 (*m*/*z* 817.5 > 255.2/113). Retention times were: OA (3.94 min), DTX1 (4.46 min), DTX2 (4.09 min). For the calibration curve, eight different concentrations of the standard (Laboratorios Cifga, Lugo, Spain) were injected in triplicate: OA/DTX1/DTX2 from 1.56 ng/mL to 100 ng/mL (Figure 4). All toxins were quantified, using their peak areas to calculate amounts and using the curve obtained from each standard. The LOD and LOQ for OA/DTX1/DTX2 were 0.0936 and 0.312 µg/Kg, respectively.

### 3.4. Statistical Analyses

The influence of the sampling site (Morocco, Madeira, and Azores islands) and organism type in the OA occurrence was analyzed using Generalized Linear Models (GLZ). The dependent variable was the concentration of OA in the organism’s flesh (µg·kg^−1^). The organisms were grouped according to their most distinctive taxonomical degree, which was not necessarily species since, in some cases, very similar species were sampled at the same time. The levels of the factor organism were: bivalve, sea cucumber, sea urchin, sea star, sea snail, and limpet. The data set consisted of OA concentrations found in pooled samples from each organism. Each sample corresponds to a single organism or a pool of organisms, in order to make up for 1 g of extracting biomass (please see methods). The dataset could be considered as a zero (inflated dataset) with a variance larger than the mean. These models are usually handled with Poisson or negative binomial distributions [41]. However, neither of these distributions could be used because our data are continuous. Instead, we used the approach of the gamma hurdle models [42] which use two steps to make the analysis: firstly, analyzing the presence/absence of the toxin (managed with a binomial or negative binomial distribution) and secondly, in these data, showing measurable concentrations of OA, a GLZ with gamma distribution. In addition to the analysis, including the “sampling site” and “organism” as factors, another analysis which only considers the “organism” as a factor was performed for which new organism levels that only occurred in a single sampling site were included: mussel, barnacle, sea cucumber, and sea hare. All the models were performed with R software [43], package *stats* function *glm*.

## 4. Conclusions

In this study, we used the UPLC-MS/MS technique for the screening of non-described vectors for Okadaic acid and its derivatives in the northwestern coast of Morocco, Madeira, and São Miguel Islands (Madeira and Azores archipelagos, respectively). We detected OA in a total of nine new vectors among the 22 screened species: echinoderms (*A. aranciacus*, *A. lixula*, *E. sepositus*, *H. sanctori*, *O. ophidianus*) and gastropods (*O. celtica*, *A. depilans*, *Patella* spp., and *S. haemostoma*). We also detected OA in the eggs of *S. haemostoma*. Regarding species uptake, the organisms with a higher uptake tendency were mussels, followed by gastropods and echinoderms. Due to the scarcity of mussels in Madeira and Azores archipelagos, gastropods and echinoderms could be a good alternative for the monitoring of this group of toxins. Although the detected values are below the limit value currently implemented in the European Union, it is important to extend the monitored organisms beyond bivalves to learn more about the trophic transfer of these toxins and OA seasonal dynamics to better calculate human health risk in these poorly studied areas.
